# SOX9 drives the epithelial–mesenchymal transition in non-small-cell lung cancer through the Wnt/β-catenin pathway

**DOI:** 10.1186/s12967-019-1895-2

**Published:** 2019-05-06

**Authors:** Jing-Qiang Huang, Fa-Kai Wei, Xiu-Li Xu, Shi-Xing Ye, Jun-Wei Song, Pei-Kun Ding, Jing Zhu, He-Feng Li, Xin-Ping Luo, Hui Gong, Li Su, Lin Yang, Li-Yun Gong

**Affiliations:** 1 Guangdong Key Laboratory for Genome Stability and Human Disease Prevention, Department of Biochemistry and Molecular Biology, Health Science Center, Shenzhen University, Shenzhen, 518060 People’s Republic of China; 2 Department of Equipment, Shenzhen Longhua People’s Hospital, Shenzhen, 518109 People’s Republic of China; 3 Department of Thoracic Surgery, Shenzhen People’s Hospital, 2nd Clinical Medical College of Jinan University, Shenzhen, 518020 People’s Republic of China; 4 Central Laboratory, Shenzhen Nanshan People’s Hospital, Shenzhen University, Shenzhen, 518052 People’s Republic of China; 5 Key Laboratory of Molecular Biophysics of Ministry of Education, College of Life Science and Technology, Huazhong University of Science and Technology, Wuhan, 430074 China; 6 Institute of Huazhong, University of Science and Technology in Shenzhen, Shenzhen, 518063 China; 7 School of Biomedical Engineering, Health Science Center, Shenzhen University, Shenzhen, 518060 People’s Republic of China

**Keywords:** SOX9, NSCLC, Metastasis, EMT, Wnt/β-catenin

## Abstract

**Background:**

The distant metastasis of cancer cells is a risk factor for tumor lethality and poor prognosis in non-small-cell lung carcinoma (NSCLC). Increased SOX9 expression has been associated with clinical stage and poor prognosis in NSCLC, but the molecular mechanisms by which SOX9 promotes metastasis in NSCLC are still unknown.

**Methods:**

The relationship between SOX9 expression and T, N, M classification was assessed using the χ^2^ test and Spearman’s analysis in 142 immunohistochemically diagnosed specimens of NSCLC. We also generated SOX9-overexpression and SOX9-knockdown cells lines and their corresponding control cell lines by transfection with lentiviral constructs. In vivo assay, SOX9-overexpressing and SOX9-knockdown NSCLC cells were injected in zebrafish to examine distance metastasis. Gene set enrichment analysis (GSEA) was applied to analysis the correlation between SOX9 overexpression and Wnt/β-catenin pathway. Luciferase assay was used to check transcriptional activity of TCF/LEF and western blot and immunofluorescence was employed to detect β-catenin translocation in SOX9-overexpression, SOX9-knockdown and their corresponding control cell lines.

**Results:**

We found that SOX9 overexpression correlates with the T, N and M stage significantly (*p* = 0.03, 0.000, and 0.032 respectively) in 142 immunohistochemically diagnosed specimens of NSCLC. SOX9 overexpression was found to decrease the expression of the epithelial cell markers E-cadherin and γ-catenin and increase the expression of the mesenchymal cell markers N-cadherin and vimentin. An in vivo assay showed distant metastasis of the SOX9-overexpressing cells, which was not observed in the SOX9-knockdown cells. These findings indicate that SOX9 promotes distant metastasis by promoting EMT in NSCLC cells. GSEA showed that SOX9 overexpression was significantly correlated with the Wnt/β-catenin pathway which was corroborated by the expression of EMT-associated proteins in this pathway and its downstream target genes. SOX9 overexpression was also found to enhance the transcriptional activity of TCF/LEF, promote the nuclear translocation of β-catenin and increase the phosphorylation of GSK3β at Ser9. Further, inhibition of β-catenin suppressed the metastasis-promoting effects of SOX9 overexpression.

**Conclusions:**

This study is the first to report that SOX9 is associated with clinical TNM stage and indicates that SOX9 promotes migration, invasion and the EMT process through the Wnt/β-catenin pathway.

## Background

According to the Cancer Registration Report released by the National Central Cancer Registry of China in 2015, there are ~ 3.1 million newly diagnosed cancer patients and ~ 2 million cancer-associated deaths in China each year [1]. Lung cancer has the highest incidence and mortality rate among all tumors, and non-small cell lung cancer (NSCLC) is the most common lung cancer type, accounting for 80% of lung cancer-associated deaths [2]. Local cancer recurrence and distant metastases are the major causes of 90% deaths, and they are also the main prognostic factors in patients with NSCLC. Unfortunately, the majority of NSCLC patients are diagnosed with terminal-stage cancer upon hospital admission and are insensitive to chemotherapy. Due to the heterogeneous nature of the cancer, the causes of distant metastasis remain unclear. From the viewpoint of early diagnosis and timely treatment of NSCLC, it is important to understand the causes and mechanisms of distant metastasis, as well as the molecules involved.

The transcription factor SOX9 (SRY-related high mobility group-box 9) belongs to the SOX protein family, which includes SOX8, SOX9, SOX10 and SOX E [3]. SOX9 is evolutionarily conserved across many species and has essential functions in mediating embryonic development and sex determination [4]. Many basic and clinical studies have associated SOX9 overexpression with tumor malignancy and tumorigenesis in lung, colorectal, prostate and breast cancer [5–9]. For example, SOX9 knockout was found to decrease lung cancer tumorigenesis, whereas exogenous SOX9 overexpression enhanced tumor volume in mice [6]. Further, SOX9 upregulation enhances breast cancer cell proliferation in highly metastatic breast cancers [10] and is an independent marker of poor prognosis and a low 5-year overall survival rate [11, 12]. Our previous research found that the overexpression of SOX9 is associated with clinical stage and poor survival in NSCLC patients [11]. With regard to the underlying mechanism, it was found that activation of cGK-II (a cGMP-dependent protein kinase) led to the phosphorylation and inhibition of the SOX9 protein, which in turn suppressed AKT phosphorylation and arrested cultured glioma cells in the G1 phase; siRNA-induced suppression of SOX9 was also found to reduce glioma cell proliferation [13]. These data indicate that SOX9 is a potential biomarker for early stage diagnosis and may be a key point of clinical intervention in NSCLC. However, the biological functions and mechanism of action of SOX9 in promoting invasion and metastasis in NSCLC remain unclear.

The overexpression of SOX9 has also been detected in the lymph nodes of prostate cancer xenografts, in which SOX9 was found to enhance cellular growth, angiogenesis and invasion by promoting epithelial cells to expand into mesenchyme cells during fetal prostate development [7]. Epithelial–mesenchymal transition (EMT) is known as a key indicator of the early stage in the NSCLC metastasis process. Thus, the role of SOX9 in promoting EMT might be a good starting point to investigate the mechanisms via which it may influence metastasis in NSCLC, and this was the aim of the present study.

Here, we report that SOX9 overexpression decreased the protein level of epithelial cell markers and increased the protein level of mesenchymal cell markers in NSCLC cell lines, thus confirming the influence of SOX9 on EMT. The findings were corroborated by in vivo experiments in zebrafish models, in which SOX9 overexpression was found to promote distant metastasis. Further investigation into the underlying mechanism revealed that the Wnt/β-catenin pathway was involved in the metastasis-promoting effects of SOX9. In our previous study, we found that SOX9 was a predictor of poor prognosis and over expression of SOX9 had a significant correlation with the early histological stage subgroup (stages I and II) and the late histological stage subgroup (stages III and IV) in 142 NSCLC tissue sections [11]. To our knowledge, this study is the first to report that SOX9 is associated with clinical TNM stage in NSCLC, and to describe the mechanism by which SOX9 enhances Wnt/β-catenin pathway via promoting GSK3β phosphorylation at serine 9. Moreover, our findings support a significant role of SOX9 in facilitating migration, invasion, and the EMT process through the Wnt/β-catenin pathway and provide a molecular understanding of the involvement of SOX9 in NSCLC metastasis which indicates that SOX9 may be an important biomarker in NSCLC progression.

## Methods

### Patients and tissue specimens

This study was conducted on a total of 142 paraffin-embedded lung cancer specimens, which were obtained from patients who were diagnosed histopathologically at the Second Affiliated Hospital of Guangzhou Medical College between 2006 and 2010 [11]. Prior patient consent and approval from the Institutional Research Ethics Committee were obtained for the use of the clinical materials for research. The tumors were staged according to the 7th edition of the Cancer Stage Manual of the American Joint Committee on Cancer [14].

### Cell lines, culture conditions and reagents

Lung cancer cell lines A549 and NCI-H460 were purchased from the American Type Culture Collection (Manassas, VA, USA) and cultured in the Dulbecco’s modified Eagle medium (DMEM; Invitrogen, Carlsbad, USA) with 10% fetal bovine serum (FBS, Invitrogen) and 1% penicillin/streptomycin (Gibco, USA) at 37 °C in a 5% CO_2_ atmosphere, and 0.25% trypsin (Gibco, USA) was used to passage the cell lines till they reached 90% confluence. The β-catenin inhibitor XAV-939 was purchased from Selleck (S1180; Houston, TX).

### Plasmids, retroviral infection, and transfection

Human SOX9 cDNA was amplified by PCR and cloned into the pSin-EF2 lentiviral vector. Retroviral production and infection were performed as described previously [15]. Stable cell lines that expressed SOX9 were selected for 10 days with 0.5 mg/ml puromycin. We constructed stable SOX9-overexpressing cell lines A549-SOX9 and NCI-H460-SOX9, and their corresponding stable control cell lines A549-pSin-Vector and NCI-H460-pSin-Vector respectively. shRNA-mediated interference was used to create SOX9-knockdown A549 and NCI-H460 cells by transfection with lentiviral constructs expressing shRNAs against SOX9 (Ruibo, China) or control shRNA for 24 h. Cells that were positive for SOX9 knockdown were selected with 0.5 mg/ml puromycin for 10 days. We then constructed stable SOX9-knockdown cell lines A549-SOX9 sh1# and NCI-H460-SOX9 sh1# and their corresponding stable control cell lines A549-pSuper-Vector and NCI-H460-pSuper-Vector respectively.

### RNA extraction and real-time reverse transcription-polymerase chain reaction

Total RNA was extracted from cultured cells with Trizol reagent (Invitrogen), and the RNA was purified using a PureLink RNA mini kit (Invitrogen) according to the manufacturer’s instructions. Real-time reverse transcription-polymerase chain reaction (RT-PCR) was used to quantify Wnt/β-catenin pathway downstream target gene mRNA levels in A549-SOX9, NCI-H460-SOX9, A549-SOX9 sh1# and NCI-H460-SOX9 sh1# cells, as well as their corresponding control cells. Primers and probes for SOX9 and glyceraldehyde-3-phosphate dehydrogenase (GAPDH) were designed using the Primer Express software, version 5.0 (Applied Biosystems, Foster City, CA, USA). Relative fold expression was calculated with the comparative threshold cycle (2^−ΔΔCt^) method.

The sequences of the primers used were as follows:

SOX9 Forward: 5ʹ-CAAGAAGGACCACCCGGATT-3ʹ

SOX9 Reverse: 5ʹ-AAGATGGCGTTGGGGGAGAT-3ʹ

Axin2 Forward: 5ʹ-CAACACCAGGCGGAACGAA-3ʹ

Axin2 Reverse: 5ʹ-GCCCAATAAGGAGTGTAAGGACT-3ʹ

Slug Forward: 5ʹ-CGAACTGGACACACATACAGTG-3ʹ

Slug Reverse: 5ʹ-CTGAGGATCTCTGGTTGTGGT-3ʹ

Twist Forward: 5ʹ-GCCTAGAGTTGCCGACTTATG-3ʹ

Twist Reverse: 5ʹ-TGCGTTTCCTGTTAAGGTAGC-3ʹ

Snail Forward: 5ʹ-TCGGAAGCCTAACTACAGCGA-3ʹ

Snail Reverse: 5ʹ-AGATGAGCATTGGCAGCGAG-3ʹ

CDH1 Forward: 5ʹ-ATTTTTCCCTCGACACCCGAT-3ʹ

CDH1 Reverse: 5ʹ- TCCCAGGCGTAGACCAAGA-3ʹ

MMP9 Forward: 5ʹ-TGTACCGCTATGGTTACACTCG-3ʹ

MMP9 Reverse: 5ʹ-GGCAGGGACAGTTGCTTCT-3ʹ

GAPDH Forward: 5ʹ-GACTCATGACCACAGTCCATGC-3ʹ

GAPDH Reverse: 5ʹ-AGAGGCAGGGATGATGTTCTG-3ʹ

### Western blotting

Western blotting was performed as previously described [15]. Briefly, protein concentrations were determined by the BCA assay (Pierce, Rockford, USA), and equal amounts of protein were separated on 10% sodium dodecyl sulfate–polyacrylamide gels and then transferred onto polyvinylidene fluoride membranes (Millipore, Bedford, USA). The membranes were probed with a primary antibody and a secondary antibody. Expression was determined by using the Super Signal West Pico Chemiluminescent Substrate (#34580; Thermo Fisher Scientific, USA) according to the manufacturer’s instructions. The membranes were stripped and re-probed with anti-β-tubulin antibody as a loading control. The antibodies used included: anti-SOX9 (1:5000, #ab182579; Abcam), anti-E-cadherin (1:1000, #610181; BD Biosciences), anti-γ-catenin (1:1000, #610254; BD Biosciences), anti-N-cadherin (1:1000, #610920; BD Biosciences), anti-vimentin (1:1000, #550513; BD Biosciences), anti-β-catenin (1:1000, #610154; BD Biosciences), anti-GAPDH (1:1000, sc-47724; Santa Cruz Biotechnology), anti-GSK-3β (1:1000, #12456; CST), anti-GSK-3β pSer9 (1:1000, #5558; Cell Signaling Technology), anti-β-tubulin (1:5000, #2146; Cell Signaling Technology), anti-H2A (1:1000, #12349; Cell Signaling Technology), goat anti-mouse secondary antibody (1:5000, #DC02L; Merck), and goat anti-rabbit secondary antibody (1:5000, #31460; Thermo Fisher Scientific, USA).

### Transwell assay

Cell migration was analyzed in vitro using 8.0 μm Transwell Permeable Supports (Corning, New York, USA). Briefly, 5  ×  10^4^ A549-pSin-Vector, A549-SOX9, NCI-H460-pSin-Vector, NCI-H460-SOX9, A549-pSuper-Vector, A549-SOX9 sh1#, NCI-H460-pSuper-Vector and NCI-H460-SOX9 sh1# cells in serum-free medium (100 μl) were seeded into the upper part of a Transwell chamber. The lower chamber was supplemented with 650 μl DMEM medium containing 20% FBS, and the chamber was incubated at 37 °C for 24 h. Cells on the upper side of the membrane were removed with cotton swabs, and cells on the lower side of the membrane were fixed with methanol for 10 min and then stained with crystal violet for 5 min. After the membrane was washed with distilled water, the cells that had invaded the membrane were counted in three randomly chosen fields of view per Transwell, and images were captured under an Olympus^®^ CKX53 microscope. A similar procedure using a Transwell insert pre-coated with 80 μl Matrigel was performed to measure cell invasion ability in three independent assays. Comparisons between each two corresponding groups were made by two-tailed paired Student’s *t* test. Differences were considered statistically significant at *p* < 0.05 and three independent experiments were performed.

### Wound healing assay

A549-pSin-Vector, A549-SOX9, NCI-H460-pSin-Vector, NCI-H460-SOX9, A549-pSuper-Vector, A549-SOX9 sh1#, NCI-H460-pSuper-Vector and NCI-H460-SOX9 sh1# cells at a density of 1 × 10^6^ were seeded into six-well plates and cultured in DMEM containing 10% FBS to 90% confluence. The confluent cell monolayer was wounded using a sterile 200-μl pipette tip, and the cells in suspension were washed in normal growth medium. Images of the monolayer wound were captured after 0 h, 24 h and 48 h under an Olympus^®^ CKX53 microscope in three randomly chosen fields of view. The migratory ability of the cells was calculated as the ratio of the open area after 24 h and 48 h to the open area at 0 h. Comparisons between each two corresponding groups were made by two-tailed paired Student’s *t*-test. Three independent assays were performed. Differences were considered statistically significant at *p* < 0.05.

### Zebra fish assay

Transgenic Tg (flil: EGFP) zebrafish (provided by the Institute of Hydrobiology, Chinese Academy of Sciences, Wuhan, China) were maintained at 28 °C in aquaria under a 14 h/10 h day/night cycle. A549-pSin-Vector, A549-SOX9, NCI-H460-pSin-Vector, NCI-H460-SOX9, A549-pSuper-Vector, A549-SOX9 sh1#, NCI-H460-pSuper-Vector and NCI-H460-SOX9 sh1# cells were labeled with DiI stain. A total of 500 cells of each cell line were injected into the perivitelline space of 48-h post-fertilization embryos using a micro-injector (World Precision Instruments Inc., Sarasota, FL, USA). The embryos were incubated at 28 °C for 3 days before live images were captured under an Olympus^®^ BX51 microscope. The number of disseminated foci from the tumor mass in the zebrafish embryo tails was counted. Comparisons between each two corresponding groups were made by two-tailed paired Student’s *t*-test. Differences were considered statistically significant at *p* < 0.05. Five zebrafish were used in each experiment and three independent experiments were performed.

### Immunofluorescence assay

Cells (A549-pSin-Vector, A549-SOX9, NCI-H460-pSin-Vector, NCI-H460-SOX9, A549-pSuper-Vector, A549-SOX9 sh1#, NCI-H460-pSuper-Vector and NCI-H460-SOX9 sh1#) for immunofluorescence staining were grown and treated in chamber slides, fixed in 4% formaldehyde in phosphate-buffered saline (PBS) for 10 min, permeabilized for 10 min with 0.2% Triton X-100 in PBS, and blocked with 2% bovine serum albumin (BSA) for 1 h. Primary antibodies against SOX9 (#ab182579, Abcam), E-cadherin (#610181, BD Biosciences), γ-catenin (#610254, BD Biosciences), N-cadherin (#610920, BD Biosciences), vimentin (#550513, BD Biosciences), and β-catenin (#610154, BD Biosciences) were diluted to 1:400 in PBS containing 1% BSA and incubated for 1 h at room temperature. Secondary antibody was purchased from Life Technologies^®^ (Grand Island, NY), diluted to 1:250 in 1% BSA in PBS and incubated for 1 h. Images were captured with the Nikon^®^ TS2 microscope.

### Dual-luciferase reporter assay

Cells (A549-pSin-Vector, A549-SOX9, NCI-H460-pSin-Vector, NCI-H460-SOX9, A549-pSuper-Vector, A549-SOX9 sh1#, NCI-H460-pSuper-Vector and NCI-H460-SOX9 sh1#) were plated in 100-mm cell culture dishes, until they proliferated to 60–80% confluence after 24 h of culture. TOP flash or FOP flash and Renilla pRL-TK plasmids were transfected into the cells with Lipofectamine 3000 (Life Technologies) according to the manufacturer’s protocol. After 48 h of incubation, the transfection medium was replaced, and the cells were harvested, washed with PBS and lysed with passive lysis buffer (Promega). The cell lysates were analyzed immediately using a 96-well plate luminometer (Biotech, Germany). Luciferase and Renilla luciferase activity were measured using a dual-luciferase reporter assay system (Promega) according to the manufacturer’s instructions. The luciferase activity of each lysate was normalized to Renilla luciferase activity. The relative transcriptional activity was converted into fold induction above the vehicle control value [15].

### Bioinformatics analysis

The gene set enrichment analysis was performed according to a standard protocol as described previously [16, 17]. We first prepared four date files, an expression dataset file, phenotype labels file, gene sets file, and chip annotations file. Next, Input data to GSEA software and run the gene set enrichment analysis. To explore the potential pathways that may be involved in the metastatic effect of SOX9, the gene set enrichment analysis (GSEA, http://software.broadinstitute.org/gsea/index.jsp) software program was used to analyze the GEO database (GEO/GSE42127, http://software.broadinstitute.org/gsea/msigdb/index.jsp), which is the transcription profiling by array of NSCLC patients according to a standard protocol.

### Statistical analysis

All statistical analyses were carried out using the statistical software package SPSS, version 22.0 (IBM SPSS, Chicago, USA). Comparisons between groups were performed using the two-tailed paired Student’s *t*-test. The relationship between SOX9 expression and TNM classification was assessed using the χ^2^ test. Bi-variate correlations between variables were calculated using Spearman’s rank correlation coefficients. A p-value of less than 0.05 was considered to indicate statistical significance in all cases.

## Results

### Correlation of SOX9 overexpression with NSCLC TNM stage

**Fig. 1:**
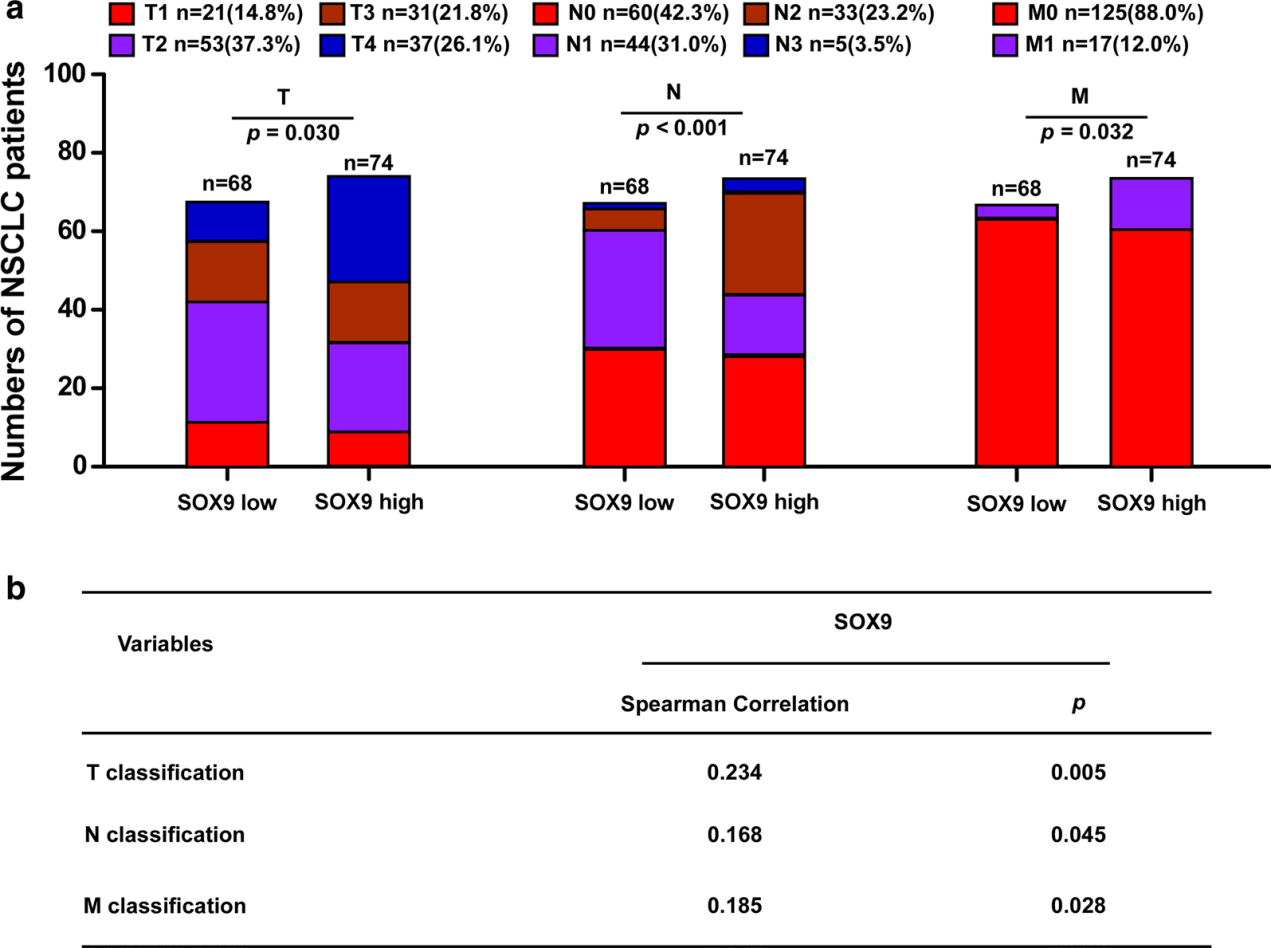
SOX9 overexpression correlates with NSCLC TNM stage significantly. **a** Correlation between TNM classification and expression of SOX9. **b** Spearman correlation analysis between SOX9 and TNM classification

We previously performed immunohistochemical staining with an antibody against human SOX9 and reported that SOX9 overexpression is significantly correlated to NSCLC histological stage (*p *= 0.017) and poor prognosis (*p *< 0.001) [11]. Here, we investigated whether the SOX9 protein expression levels also correlate with NSCLC TNM stages. We classified the 142 paraffin-embedded, archived NSCLC clinical specimens according to TNM stage (Table 1 and Fig. 1a) and found that SOX9 expression levels were positively associated with the T (*p *= 0.030), N (*p *= 0.000), and M (*p *= 0.032) stages (Table 2 and Fig. 1a). Spearman rank analysis confirmed that there was a significant correlation between increasing SOX9 expression levels and progression from the T to the N and then the M stage (Table 3 and Fig. 1b). These results indicate that NSCLC metastasis is significantly associated with SOX9 overexpression.

**Table 1:** TNM classification in 142 NSCLC patients

	No.	(%)
Classification		
T_1_	21	(14.8)
T_2_	53	(37.3)
T_3_	31	(21.8)
T_4_	37	(26.1)
N classification		
N_0_	60	(42.3)
N_1_	44	(31.0)
N_2_	33	(23.2)
N_3_	5	(3.5)
M classification		
M_0_	125	(88.0)
M_1_	17	(12.0)

**Table 2:** Correlation between TNM classification and expression of SOX9

Characteristics	SOX9		*P*-value
	Low or none	High	
T classification			
T1	12	9	0.030
T2	30	23	
T3	16	15	
T4	10	27	
N classification			
N0	31	29	0.000
N1	29	15	
N2	6	27	
N3	2	3	
M classification			
M_0_	64	61	0.032
M_1_	4	13

**Table 3:** Spearman correlation analysis between SOX9 and TNM classification

Variables	SOX9	
	Spearman correlation	P-value
T classification	0.234	0.005
N classification	0.168	0.045
M classification	0.185	0.028

### Effect of SOX9 on the migration and invasion ability of NSCLC cell lines

**Fig. 2:**
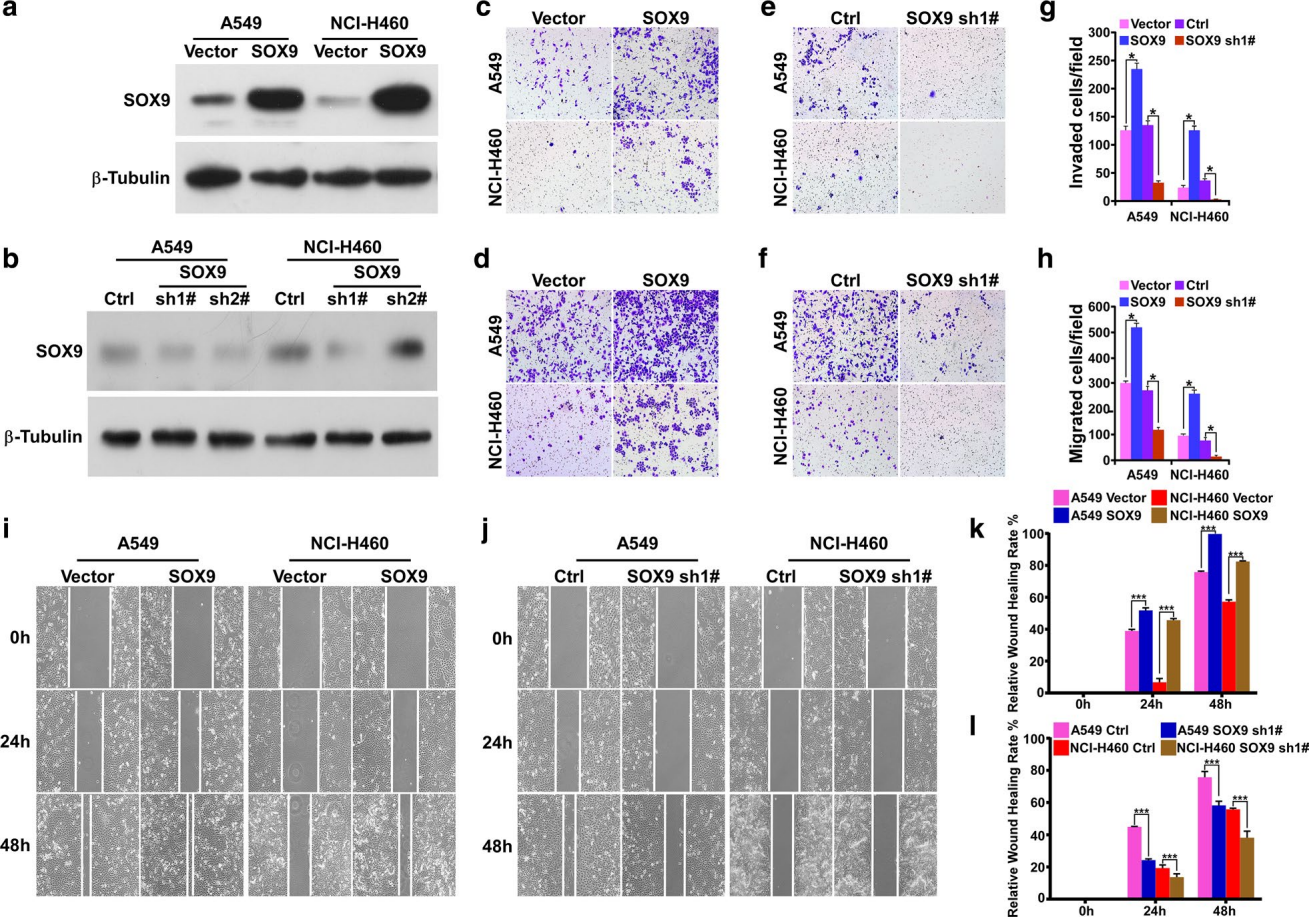
SOX9 promotes NSCLC cell invasion and migration. **a** SOX9-overexpression cell lines were analyzed by western blotting for SOX9 expression. β-tubulin was used as a loading control. **b** SOX9-knockdown cell lines were analyzed by western blotting for SOX9 expression. β-tubulin was used as a loading control. **c**, **e** Invasion assay with the Trans-well chamber and Matrigel layer was performed with the SOX9-overexpression and SOX9-knockdown cell lines. The number of cells that passed through the bottom of the Trans-well chamber with the Matrigel layer was counted, as shown in **g**, *p < 0.05. **d**, **f** Migration assay with the Trans-well chamber without the Matrigel layer was performed on the SOX9-overexpression and SOX9-knockdown cell lines. The number of cells that passed through the bottom of the Trans-well chamber without the Matrigel layer was counted as shown in **h**, *p < 0.05. **i**, **j** Wound healing assay was employed to detect the cell migration ability of SOX9-overexpressing and SOX9-knockdown cells. **k, l** Quantify the wound healing assay from **i** and **j**, ***p < 0.0001

To investigate the role of SOX9 played in NSCLC metastasis, we transduced A549 and NCI-H460 NSCLC cells with a pSin/SOX9-derived retroviral construct to establish stable SOX9-over-expressing cell lines (Fig. 2a) or pSuper/SOX9 shRNA-derived retroviral construct to establish SOX9-knock down cell lines (Fig. 2b).

A Transwell migration assay was used to determine the invasive and migratory ability of the SOX9-overexpressing cell lines A549-SOX9 and NCI-H460-SOX9 and the SOX9-knockdown cell lines A549-SOX9 sh1# and NCI-H460-SOX9 sh1#, as well as their corresponding control cells. The assay results showed that overexpression of SOX9 resulted in an increase in the number of cells that passed through the bottom of the Transwell chamber with or without the Matrigel layer, as compared to the control groups. Downregulation of SOX9 had converse effects. These data indicate that the invasive and migratory capacity of SOX9-overexpressing cells was enhanced, while the capacity of SOX9-knockdown cells was reduced in comparison to the control groups (Fig. 2c–h).

The wound healing assay was employed to detect the cell migration ability, and the results also showed that SOX9 overexpression could accelerate the migration rate as compared to the control cells (Fig. 2i–l). Contrasting results were obtained in the SOX9-knockdown cell lines as compared to the control cells.

Taken together, our data indicate that overexpression of SOX9 could improve the invasion ability of NSCLC cells and thereby promote their transformation into the malignant phenotype.

### In vivo and in vitro effect of SOX9 on inducing EMT and promoting distant metastasis

**Fig. 3:**
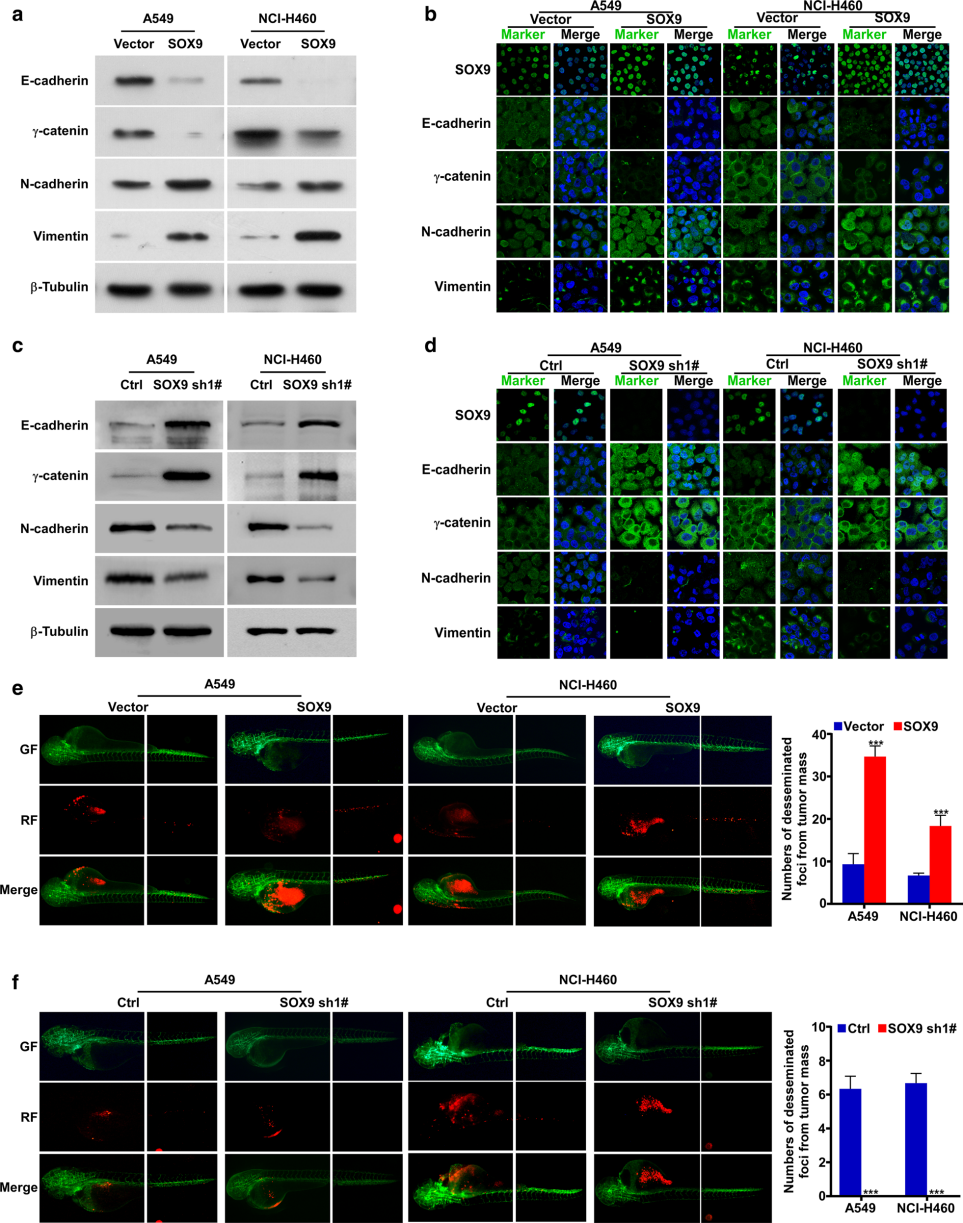
SOX9 induces EMT and promotes distant metastasis in NSCLC cells and a zebrafish model of NSCLC. **a**, **b** Western blotting and immunofluorescence analyses were used to determine the protein level of epithelial cell markers (E-cadherin and γ-catenin) and mesenchymal cell markers (vimentin and N-cadherin) in A549-SOX9 and NCI-H460-SOX9 cell lines and their corresponding control A549-Vector and NCI-H460-Vector cells. **c**, **d** Western blotting and immunofluorescence analyses were used to determine the protein level of epithelial cell markers (E-cadherin and γ-catenin) and mesenchymal cell markers (vimentin and N-cadherin) in A549-SOX9 sh1# and NCI-H460-SOX9 sh1# cells and their corresponding control A549-pSuper-Vector and NCI-H460-pSuper-Vector cells. **e**, **f** Zebrafish embryos injected with either SOX9-overexpression cells or SOX9-knockdown cells were imaged live under an Olympus^®^ BX51 microscope. SOX9-overexpressing cells and SOX9-knockdown cells are labeled with red fluorescence and zebrafish are labeled with green fluorescence. Quantification of the number of disseminated tumor foci (n = 5 per group) in the tail regions of Zebrafish embryos, ***p < 0.0001

Western blotting and immunofluorescence analyses revealed that expression of the epithelial cell markers (E-cadherin and γ-catenin) was downregulated and expression of the mesenchymal cell markers (vimentin and N-cadherin) was upregulated at the protein level in the SOX9-overexpressing cell lines as compared to the corresponding control cell lines (Fig. 3a, b). In contrast, expression of the epithelial cell markers (E-cadherin and γ-catenin) was upregulated and expression of the mesenchymal cell markers (vimentin and N-cadherin) was downregulated at the protein level in the SOX9-knockdown cells as compared to the corresponding control cells (Fig. 3c, d).

Next, we examined the in vivo effect of SOX9 over-expression on metastasis in a zebrafish model. We microinjected each constructed cell line and after 1 week found increased numbers of SOX9-over-expressing disseminated tumor foci (A549-SOX9 and NCI-H460-SOX9) in the zebrafish tail compared to the control groups (A549-pSin-Vector and NCI-H460-pSin-Vector). Conversely, we found decreased numbers of SOX9-knockdown disseminated tumor foci (A549-SOX9 sh1# and NCI-H460-SOX9 sh1#) in the zebrafish tail compared to the control group (Fig. 3e, f). These results indicate that SOX9 over-expression can promote in vivo distant metastasis of NSCLC.

### Promotion of EMT by SOX9 via activation of the Wnt/β-catenin pathway in NSCLC cell lines

**Fig. 4:**
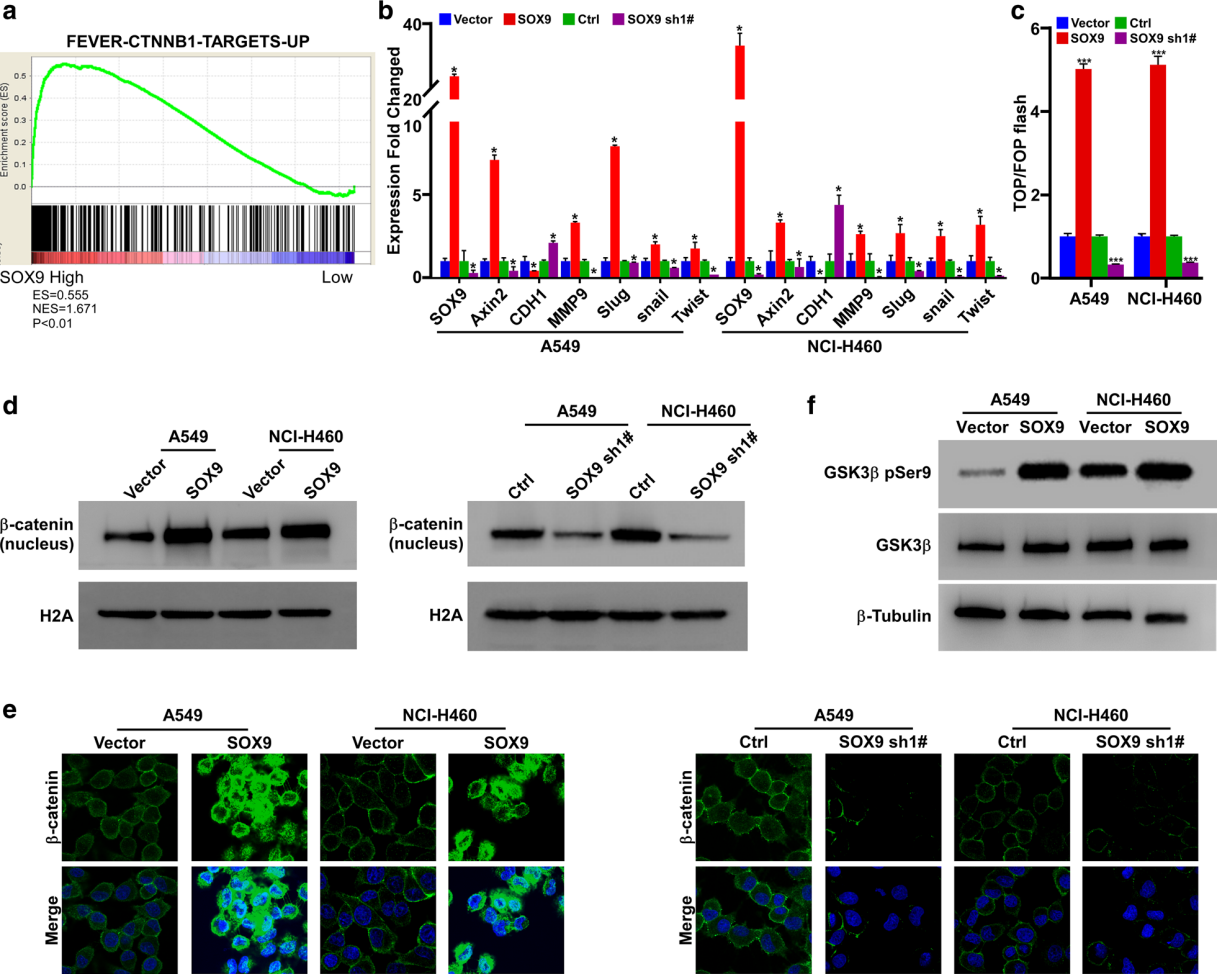
SOX9 promotes EMT by enhancing β-catenin nuclear translocation and TCF1/LEF1 transcriptional activity, and thereby activating the Wnt/β-catenin pathway in NSCLC cell lines. **a** GSEA correlating SOX9 levels with Wnt/β-catenin pathway genes on publicly available NSCLC patient gene expression profiles (NCBI/GEO/GSE42127, http://software.broadinstitute.org/gsea/msigdb/index.jsp). **b** Real-time PCR analysis of metastasis-associated gene in the indicated cells, *p < 0.05. **c** Dual luciferase reporter assay showing TCF1/LEF1 transcriptional activity. Reporter activity was normalized to that of Renilla luciferase activity, ***p < 0.0001. **d** Western blot of β-catenin in the nuclear fractions of the indicated cells. **e** Immunofluorescence representative of subcellular β-catenin localization in the indicated cells. **f** Western blot of the GSK3β and GSK3β pSer9 in the indicated cells. β-Tubulin was used as a loading control

To investigate the mechanism of SOX9-induced EMT, we performed gene enrichment analysis with the GSEA software. We found that SOX9 was significantly associated with the Wnt/β-catenin signaling pathway (Fig. 4a). Therefore, RT-PCR analysis was used to quantify the mRNA levels of genes involved in the Wnt/β-catenin signaling pathway that are associated with metastasis, invasion, migration and EMT. Further, the RNA expression levels of downstream target genes of the Wnt/β-catenin pathway were detected by real-time PCR. The data showed that CDH1 (E-cadherin) expression was significantly decreased, and that Twist, snail, MMP9, Axin2, and Slug expression was significantly increased in SOX9-overexpressing cells as compared to the corresponding control cells. We also found that the expression of Twist, snail, MMP9, Axin2 and Slug was significantly decreased and the expression of CDH1 (E-cadherin) was significantly increased in the SOX9-knockdown cells as compared to the corresponding control cells (Fig. 4b).

In the classical Wnt/β-catenin pathway, β-catenin accumulates in the nucleus and binds to TCF1/LEF1 to promote downstream target gene expression. We therefore performed a dual luciferase reporter assay to detect TCF1/LEF1 transcriptional activity. The luciferase fluorescence signal versus Renilla fluorescence signal was increased in the SOX9-overexpressing A549-SOX9 and NCI-H460-SOX9 cells but was decreased in the SOX9-knockdown A549-SOX9 sh1# and NCI-H460-SOX9 sh1# cells, as compared to the corresponding control cells. Thus, upregulation of SOX9 expression enhanced TCF1/LEF1 transcriptional activity (Fig. 4c). This means that SOX9 might activate the Wnt/β-catenin pathway by promoting β-catenin translocation.

Next, we analyzed β-catenin protein expression in nuclear extracts from SOX9-overexpressing cells and SOX9-knockdown cells. The β-catenin expression in the nucleus was increased in SOX9-overexpressing cells and decreased in SOX9-knockdown cells (Fig. 4d). Further, immunofluorescence assay showed enhanced β-catenin translocation from the cytoplasm to the nucleus in the SOX9-overexpressing cells but not in the SOX9-knockdown cells (Fig. 4e). This finding indicates that SOX9 promotes the localization of β-catenin to the nucleus, where it acts as a co-activator for enhancing the expression of downstream target genes of the Wnt/β-catenin signaling pathway.

GSK3β is a key regulatory molecule in both the Wnt/β-catenin and the phosphatidylinositol 3-kinase/AKT pathways. Akt activated by PI3 K is known to have the capacity to inactivate GSK3β by phosphorylating it at residue Ser9. Once GSK3β is phosphorylated at residue Ser9, the complex formed by GSK3β, Axin and APC is destroyed; this is the mechanism responsible for the translocation of β-catenin to the nucleus. In this study, we found that the expression of GSK-3β was not significantly changed by SOX9 overexpression; however, the phosphorylation level of GSK-3β at Ser9 was markedly increased (Fig. 4f). Thus, activation of the Wnt/β-catenin pathway by SOX9 might be initiated by the phosphorylation of GSK3β at Ser9 and the subsequent nuclear translocation of β-catenin.

### Effect of the β-catenin inhibitor XAV-939 on EMT induced by SOX9

**Fig. 5:**
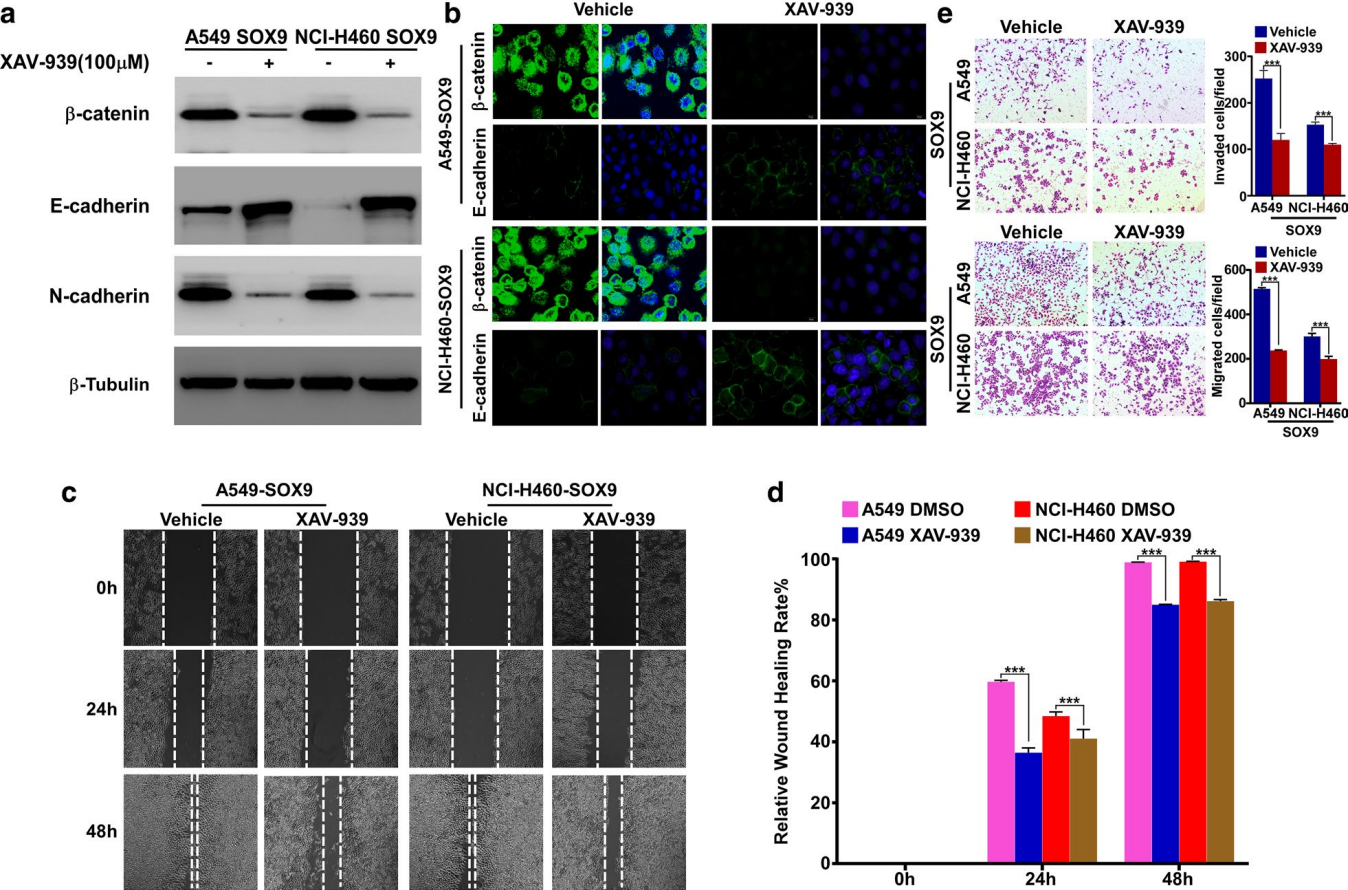
EMT induced by SOX9 could be reversed by the β-catenin inhibitor XAV-939. **a** Western blot of the β-catenin, E-cadherin and N-cadherin proteins after treatment with the β-catenin inhibitor XAV-939 (100 μM) for 48 h in the indicated cells. β-Tubulin was used as a loading control. **b** Immunofluorescence of subcellular β-catenin and E-cadherin localization after treatment with the β-catenin inhibitor XAV-939 (100 μM) for 48 h in the indicated cells. **c** Wound healing assay to detect the migration ability after treatment with the β-catenin inhibitor XAV-939 (100 μM) for 48 h in the indicated cells. **d** Quantify the wound healing assay from **c**, ***p < 0.0001. **e** The Trans-well assay with or without Matrigel was performed in SOX9-overexpression cell lines after treatment with the β-catenin inhibitor XAV-939 (100 μM) for 48 h. The number of cells that passed through the bottom chamber are quantified and shown in the right panel, ***p < 0.0001

Finally, we performed an assay to determine whether SOX9-induced EMT could be reversed by inhibiting β-catenin function with the β-catenin inhibitor XAV-939. We cultured A549-SOX9 and NCI-H460-SOX9 (SOX9-overexpression) cells in the presence of XAV-939 (100 μM) and evaluated EMT marker expression by western blotting. Inhibition of β-catenin expression was found to increase E-cadherin expression and decrease N-cadherin expression; thus, the EMT process might be reversed by the β-catenin inhibitor XAV-939 (Fig. 5a, b). The wound healing assay and Transwell assay also showed that XAV-939 application inhibits the migratory capacity of SOX9-overexpressin cells (Fig. 5c–e).

## Discussion

In the present study, we were able to confirm the role of SOX9 in the progression of NSCLC. The pathological data obtained from 142 NSCLC patients confirmed that SOX9 overexpression is significantly associated with TNM stage. We thus focused on the role of SOX9 in distant metastasis in NSCLC and its underlying molecular mechanism.

Distant metastasis is the last step in solid tumor progression and the highest risk factor for host death [18]. Distant metastasis involves the detachment of tumor cells from their primary location or tumor, their invasion into circulation or the lymphatic system, and their proliferation at a location that is distant from the primary tumor [19]. In thyroid cancer cells, knockdown of SOX9 has been found to inhibit proliferation, invasion, and EMT [20]. We thus modeled this process by microinjecting fluorescently labeled SOX9-over-expressing or SOX9-knockdown cells into zebrafish larvae. We found that zebrafish injected with SOX9-over-expressing cells developed more disseminated tumor foci and zebrafish injected with SOX9-knockdown cells developed less disseminated tumor foci than control zebrafish. These data support our hypothesis that SOX9 overexpression promotes distant metastasis in NSCLC.

EMT is an early event in tumor metastasis. It is characterized by downregulation of epithelial cell markers (E-cadherin, γ-catenin and claudin-1) and upregulation of mesenchymal cell markers (vimentin, fibronectin, and N-cadherin) [21, 22]. In a human chondrocyte cell line, miR-19a was found to promote cell viability and migration of chondrocytes via positive regulation of SOX9 expression [23]. Further, overexpression of miR-216b suppressed NSCLC cell proliferation and invasion by targeting SOX9. Interestingly, the effects of miR-216b can be mimicked by siRNA against SOX9 [24]. Here, we found that SOX9 overexpression results in an increase in N-cadherin and vimentin expression and a decrease in E-cadherin and γ-catenin expression. The opposite results are obtained in SOX9 knockdown cells. These data indicate that SOX9 overexpression may promote NSCLC metastasis by inducing EMT.

Many signaling pathways are involved in EMT-mediated metastasis, including the TGF-β, MAPK, AKT, PI3 K, Notch and WNT pathways [25–28]. It was reported recently that SOX9 participates in the feedback loop of MALAT1 (metastasis associated lung adenocarcinoma transcript 1)-miR-101-SOX9, which modulates the chemoresistance of lung cancer cells to Cisplatin via the Wnt signaling pathway [29]. In the present study, we found that SOX9 was significantly associated with the Wnt/β-catenin pathway, based on the results of GSEA with the GEO database (GEO/GSE42127). This was confirmed by the increased transcription of downstream metastasis-associated genes of the Wnt/β-catenin signaling pathway in the SOX9-overexpressing cells.

Our nuclear extraction assay and immunofluorescence analysis showed that SOX9 overexpression promoted β-catenin translocation from the cytoplasm to nucleus, but this was not observed in the SOX9-knockdown cells. This finding combined with the dual luciferase reporter assay finding that SOX9 overexpression indirectly enhanced TCF1/LEF1 transcriptional activity indicates that SOX9 promoted the translocation of β-catenin to the nucleus. Similarly, Blache et al. reported that SOX9 overexpression is the precursor of β-catenin accumulation in the nucleus, and that it acts on TCF4 to form a β-catenin–TCF4 complex in colon cancer [30]. To further verify the role of SOX9 in inducing EMT by β-catenin nuclear translocation, we used the β-catenin inhibitor XAV-939. Our data showed that SOX9-induced EMT could be reversed upon exogenous application of XAV-939. Taken together, these results demonstrate that SOX9 promotes EMT by activating the Wnt/β-catenin pathway through the nuclear translocation of β-catenin.

A study on chondroplasia showed that the N-terminal of SOX9 binds to β-catenin and induces β-catenin translocate to the nucleus. Once SOX9 induced the nuclear translocation of β-catenin, the phosphorylation and degradation of β-catenin was accelerated by GSK-3β [31]. In the NSCLC cells used in the present study, we found that the GSK-3β levels were not altered as a result of SOX9 overexpression; however, GSK-3β phosphorylation at serine 9 was significantly increased. Thus, SOX9 might promote metastasis in NSCLC by activating the Wnt/β-catenin pathway through enhancing the phosphorylation of GSK-3β at Ser9. The interaction between SOX9 and GSK-3β needs to be further investigated, especially with regard to whether SOX9 is associated closely with the reduction of β-catenin phosphorylation.

## Conclusion

Our study confirms that SOX9 promotes distant metastasis by driving the EMT in NSCLC. We show that the Wnt/β-catenin pathway is activated as a result of SOX9 promoting β-catenin translocation from the cytoplasm to the nucleus to enhance TCF1/LEF1 transcriptional activity. This effect may be initiated by SOX9-mediated phosphorylation of GSK-3β at Ser9. Further studies on the relevance of SOX9-mediated GSK3β phosphorylation at Ser9 are now required to better understand the mechanisms of SOX9. Collectively, these findings suggest that manipulation of SOX9 to impede NSCLC metastasis and malignancy may be clinically useful and provide a new insight for the intervention of distal metastasis in cancer therapy.

## Abbreviations

AKT/PKB: protein kinase B
ATCC: American Type Culture Collection
BCA: bicinchoninic acid
BSA: bovine serum albumin
cDNA: complimentary deoxyribonucleic acid
cGKII: cyclic guanosine monophosphate-dependent protein kinase II
DAPI: 4′,6-diamidino-2-phenylindole
DMEM: Dulbecco Modified Eagle Medium
DMSO: dimethyl sulfoxide
EGFR: epidermal growth factor receptor
EMT: epithelial–mesenchymal Transition
FBS: fetal bovine serum
GAPDH/G3PDH: glyceraldehyde 3-phosphate dehydrogenase
GSK-3β: glycogen synthase kinase-3β
gsea: gene set enrichment analysis
H: hour
HMG: high mobility group
LEF: lymphoid enhancer-binding factor
NSCLC: non-small cell lung cancer
PBS: phosphate buffered saline
PCR: polymerase chain reaction
PI3K: phosphatidylinositol-3 kinase
Puro.: puromycin
RNA: ribonucleic acid
RT-PCR: real-time reverse transcription-polymerase chain reaction
SCLC: small cell lung cancer
SOX9: SRY-related High Mobility Group-box 9
SRY: sex-determining region Y
TBS: tris buffered saline
TCF: T cell transcription factor
TNM: tumor node metastasis
